# Fatal Asphyxiation in Bottlenose Dolphins (*Tursiops truncatus*) from the Indian River Lagoon

**DOI:** 10.1371/journal.pone.0066828

**Published:** 2013-06-19

**Authors:** Megan Stolen, Judy St. Leger, Wendy Noke Durden, Teresa Mazza, Erika Nilson

**Affiliations:** 1 Hubbs-SeaWorld Research Institute, Melbourne Beach, Florida, United States of America; 2 SeaWorld San Diego, San Diego, California, United States of America; Università degli Studi di Napoli Federico II, Italy

## Abstract

Multiple single case reports of asphyxiation in dolphins caused by fish lodged in the esophagus exist. However, the significance of this cause of mortality in a single population has not been documented. We performed a retrospective evaluation of pathology records from stranded bottlenose dolphins (*Tursiops truncatus*) from the Indian River Lagoon to evaluate the impact of this cause of death on this population. From 1997 to 2011, asphyxiation due to choking was identified as the cause of death in 14 of 350 cases (4%). Sampling of an unrelated but adjacent population over this same period yielded 186 necropsy cases of bottlenose dolphins with no cases of asphyxiation. Asphyxiated animals presented with a fish lodged in the cranial esophagus associated with a dislocated and obstructed or compressed larynx. There was no clear sex predilection. Affected animals included 12 adults and two juveniles. The fish species involved included sheepshead, black chin tilapia and striped mojarra. In five cases, recreational fishing gear was also present. Cetacean choking is related to selection of prey fish species with strong dorsal spines and may be secondarily associated with fish attached to fishing gear. Prey abundance and dolphin behavior may influence these selections. Environmental alterations leading to changes in prey availability or increased interactions with fishing gear may change the significance of fatal choking in dolphin populations.

## Introduction

**Figure 1:**
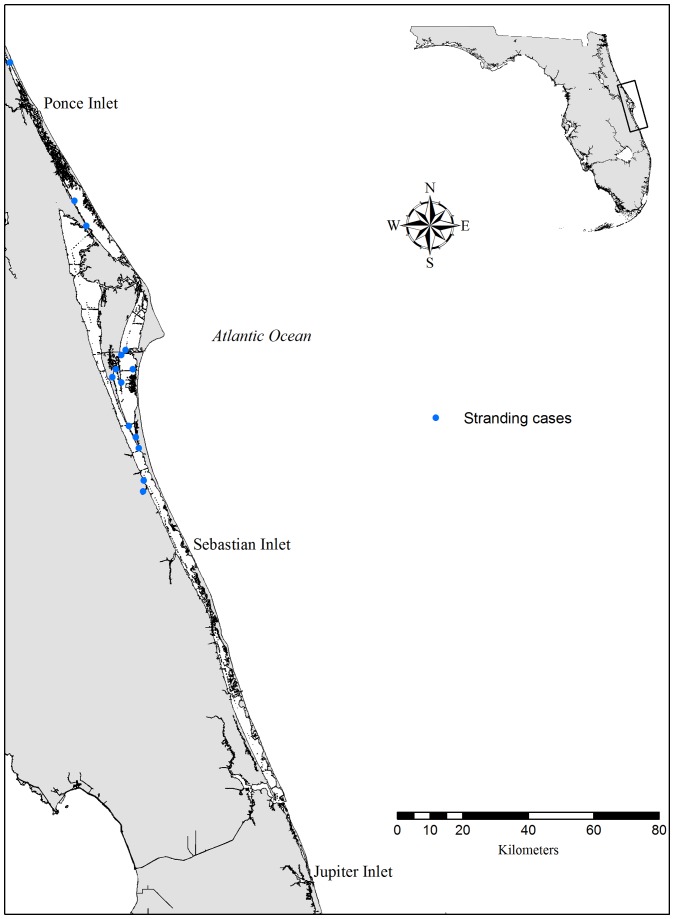
Map of study area. Study area on the central east coast of Florida. The Indian River Lagoon is comprised of three interconnected water bodies separated from the Atlantic Ocean by barrier islands. Records of stranded dolphins that died of asphyxiation are symbolized as blue dots.

The Indian River Lagoon (IRL) is a shallow estuary system that extends approximately 220 km along the east coast of central Florida [1] (Fig. 1). While the estuary is open to the Atlantic Ocean at five inlets, there is a population of bottlenose dolphins (*Tursiops truncatus*) within the lagoon that demonstrates high site fidelity. These animals comprise a defined stock [2] and are monitored regularly within the lagoon.

Fatal asphyxiation (choking) in bottlenose dolphins resulting from fish lodged in the esophagus has been reported in several previous cases [3], [4], [5], [6]. However, multiple cases from the sample geographic area have not been previously reported. The current study presents fourteen cases in the IRL population over a fifteen year period and compares the incidence of this diagnosis to another population in the vicinity.

## Methods

Ethics Statement: Samples from stranded animals were collected under the authority of NOAA and the MMPA 1972 under a Stranding Agreement as part of the Marine Mammal Health and Stranding Response Act to respond to and collect samples from stranded marine mammals.

Necropsy reports from the IRL population (from Ponce Inlet to Sebastian Inlet, Fig. 1) were reviewed for cases of asphyxiation. For comparison, a second group of cases included animals stranding at the same latitude, but on the east side of the barrier islands (that enclose the IRL) along the eastern Florida coast.

All animals were examined according to established gross and histologic examination protocols. Only carcasses ranging from fresh to moderate decomposition (code 2- early code 3) were included in this study [7]. Sex was determined by external examination and examination of gonads. In one affected animal, decomposition and predation precluded determination of sex. Tissues from all major organs were collected by standardized protocols [7], fixed in 10% neutral buffered formalin, embedded in paraffin, sectioned at 3–5 *um*, and stained with hematoxylin and eosin for examination by light microscopy. Histopathologic findings were characterized as incidental, significant and/or cause of death.

Subsequent to the gross examination, fish found causing laryngeal obstruction were examined and identified based on comparison to a standard identification guide [8]. When possible, the fish were measured to determine length or length was estimated based on the available tissue.

## Results

**Figure 2:**
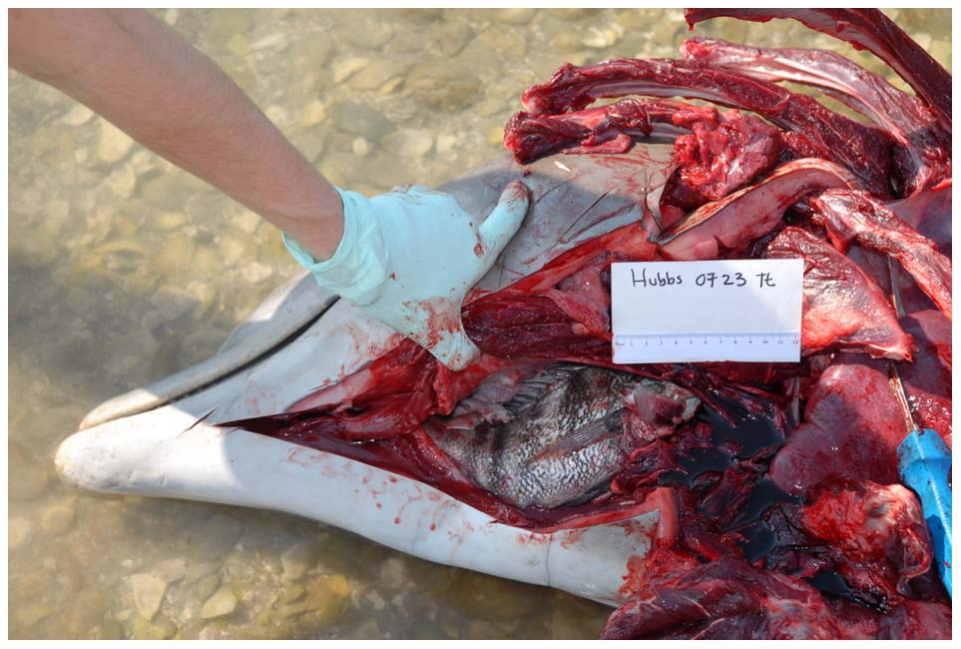
Photograph of stranded dolphin with fish. Photograph of IRL dolphin (Hubbs-0723) with sheepshead lodged in the esophagus.

**Figure 3:**
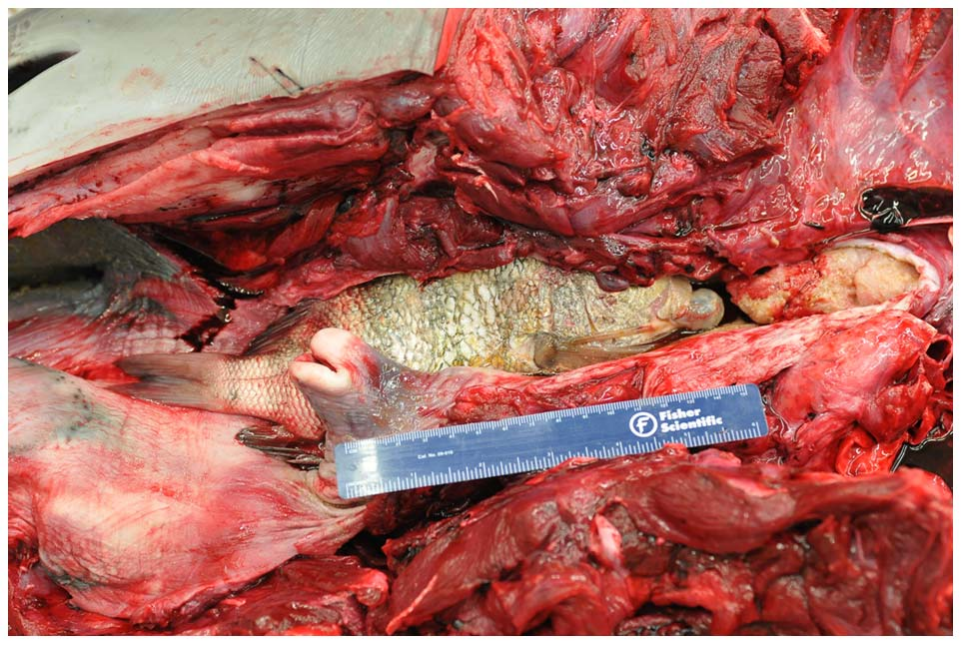
Photograph of dolphin with fish in esophagus. Photograph of IRL dolphin (Hubbs-1075) showing large fish displacing the laryngeal cartilage causing asphyxiation.

Over the study period, 350 dolphin carcasses were examined in the IRL and examined for cause of death. Fatal asphyxia was diagnosed as the cause of death when laryngeal displacement, compression, or obstruction was indentified and no significant histologic changes were identified as the cause of death. Case review demonstrated 14 cases (4%, Table 1). In at least five cases, dolphins were found with the tail of the prey fish extending from the mouth. On gross examination, all of the dolphins demonstrated fish lodged in the esophagus displacing or compressing the larynx (Fig. 2 and 3). In seven cases, fish were firmly lodged and held into the area of the larynx by dorsal spines that were embedded in the esophageal wall. Eight cases involved males, five were females, and one animal could not be clearly identified. Twelve dolphins were adults and two of the animals were juveniles. Detailed examinations of the lodged fish resulted in identification of eight of the 14 fish. Positive identification of the remaining six was hindered by decomposition or missing portions of the fish. Identified fish included six cases with sheepshead (*Archosargus probatocephalus*), one black chin tilapia (*Sarotherodon melanotheron*) and one striped mojarra (*Diapterus plumieri*). Three other fish were tentative classified as a jack (Carangidae) and two tilapia (Cichlidae) but positive species identification could not be made. The fishes measured 19 cm to 40 cm.

**Table 1:** Incidence of asphyxiation in IRL bottlenose dolphins (1997–2010).

field number	age class	total length (cm)	sex	identity of fish	size of fish	human interaction comments
Hubbs-9734-Tt	adult	238	male	sheepshead	unknown	
Hubbs-0111-Tt	adult	245	male	unknown	40 cm estimated*	fishing lure attached to fish
Hubbs-0249-Tt	adult	230	female	sheepshead	31 cm	
Hubbs-0310-Tt	adult	230	unknown	sheepshead	22 cm estimated*	fishing lure in stomach
Hubbs0512-Tt	adult	258	male	unknown	unknown	
Hubbs-0723-Tt	juvenile	205	male	sheepshead	28 cm	
Hubbs-0738-Tt	adult	234	female	possible tilapia	20 cm	
Hubbs-0749-Tt	adult	212	female	black chin tilapia	19 cm estimated*	fishing lure attached to fish
Hubbs-0753-Tt	adult	248	male	unknown	28 cm estimated*	
Hubbs-0754-Tt	adult	243	female	sheepshead	26 cm estimated*	
Hubbs-0826-Tt	adult	220	male	striped mojarra	30 cm estimated*	fishing line and hooks embedded in esophagus
Hubbs-0853-Tt	adult	278	male	possible tilapia	37 cm	entangled around flukes in fishing line
Hubbs-0955-Tt	adult	241	female	unknown	unknown	
Hubbs-1075-Tt	juvenile	192	male	sheepshead	29 cm estimated*	

* Sizes of fish were estimated when only partial remains were available.

In five of the asphyxia cases, recreational fishing gear was found associated with the fish. In two cases, lures were found attached to the fish anchoring the fish in place and in one case, monofilament line and hooks were attached to the fish and embedded in the dolphin's esophagus. In two cases, line or lures were found incidentally unrelated to the fish. For comparison, cases from the adjacent Atlantic Ocean were evaluated for incidence of choking. During the study period, 186 carcass examinations were conducted and no cases of asphyxia were found. In this population, the sex and age class distribution was similar to the IRL population. There were no animals found with recreational fishing gear.

## Discussion

In cetaceans, underwater feeding strategies necessitate a modified upper respiratory anatomy to prevent aspiration. The elongated laryngeal cartilages extend through the esophagus to insert directly into the nasal passage. Separation of the digestive and respiratory tracts is assured by the palatopharynegeal sphincter muscle [9], [10]. Prey passes on either side of the laryngeal cartilages by way of the paired piriform sinuses [11]. Mignucci-Giannoni *et al.* [5] describe this structural adaptation and argue that this mechanism “makes asphyxiation due to choking very unlikely in odonotocetes”. However, laryngeal dislocation into the esophagus can be accomplished with moderate pressure [12] and is necessary to ingest larger prey items. When the larynx is dislocated, the respiratory and digestive tracts comingle. The elongate larynx is vulnerable to compression and obstruction when within the esophageal lumen and asphyxiation and choking can occur. Thus, the unique morphology of the upper respiratory tract in odontocetes may instead make them particularly more vulnerable to esophageal obstruction leading to asphyxiation [11] especially when large or spiny fish are ingested. The peg-like anatomy of the larynx also predisposes odontocetes to serious injury when fishing gear becomes wrapped or imbedded in or around the base of the cartilages [13].

A number of single cases of fatal aspiration have been reported in the literature. A report from the Gulf of Mexico describes a 2.7 year old bottlenose dolphin found dead stranded with a 37 cm long decapitated sheepshead lodged in the esophagus. The estimate whole body length of this fish was 42 cm [6]. An adult female bottlenose dolphin was reported dead on the shore in Puerto Rico with a 49cm black margate (*Anisotremus surinamensisi*) lodged in her esophagus [5]. A young adult male Indo-pacific bottlenose dolphin (*Tursiops aduncus*) was found dead on the shore at Adeliaide, Australia with a 66 cm cobbler carpet shark (*Sutorectus tentaculatus)* lodged in the esophagus with the tail sticking out of the gape of the dolphin's mouth [3]. In the same area, another Indo-Pacific bottlenose dolphin died with a Slender-spined Porcupine Fish (*Diodon nichthemerus*) in the posterior pharynx and upper esophagus [4]. All of these cases occurred as single events and include large fish and/or fish with abundant strong spines. Two previous reports of IRL dolphins choking have been documented [14]. This study looks not only at single cases but evaluates the importance of the condition as a cause of death in a defined dolphin stock.

The fish associated with asphyxiation are likely important in understanding the prevalence of this condition. Published cases from other study sites showed that large fish were associated with laryngeal displacement [3], [5], [6]. However, the range in fish length at consumption for bottlenose dolphins in the IRL is 4.4 to 36.4 cm [15]. Fish associated with asphyxiation in the IRL had lengths well within this range so total length is likely not a factor in our study area. However, the species of fish is likely very important. The most prevalent fish (sheepshead and tilapia) associated with choking are both deep-bodied and exhibit strong dorsal spines. These spines can perforate the dolphin's esophagus causing the fish to become anchored in the esophagus so that swallowing cannot be completed. Attempts to manipulate the fish in the upper esophagus likely result in the displacement of the larynx in spite of the smaller sized fish. This can lead to asphyxiation regardless of overall fish size. In some cases, fishing gear that is attached to the fish may also hinder the dolphin's ability to completely swallow the prey fish. In these instances, hooks can perforate the esophagus or laryngeal cartilages to anchor the prey fish or line can entangle the larynx preventing replacement of the larynx back into place below the internal nares.

Sheepshead are considered native and common to abundant throughout the southeastern Atlantic states as well as on the Gulf coast and are common throughout the IRL [16], however they are not considered a common or important prey fish for bottlenose dolphins in the IRL [15], [17]. Rather, these fish may be considered a “risky” prey item for bottlenose dolphin because of their spines and may be avoided by dolphins. *Tilapia* sp. are non-native and are becoming increasingly common in the IRL [18]. These fish may represent a novel prey item to dolphins but with similar body type and strong dorsal spines. Differences in choking deaths between the two adjacent populations may be a result of differences in the prey abundance and distribution or differences in foraging ecology between dolphin groups.

If future changes in environmental conditions produce changes in fish abundance or prey preference in dolphins of the IRL, dolphins may be exposed to more risk. Likewise, as recreational gear use increases in this estuary, hooked fish may become more prevalent and increase the risk of asphyxiation. Work by Harris *et al.* [19] with several species of seabirds showed that environmental changes can lead to changes in prey fish. This shift can result in a significant change in prey quality (lower caloric value) in prey fish but was also associated with an increase in choking deaths in young birds. Optimal foraging is important across taxonomic groups and dolphins are no exception. Changes due to fishing pressure, water temperature, salinity and introduction of invasive fish species can all lead to changes in prey fish availability. This shifting baseline for prey can lead to increased risk for dolphins in populations with high site fidelity.
